# β-arrestin2 regulating β2-adrenergic receptor signaling in hepatic stellate cells contributes to hepatocellular carcinoma progression

**DOI:** 10.7150/jca.59291

**Published:** 2021-10-28

**Authors:** Xiu-Qin Li, Wen-Ting Peng, Shan Shan, Jing-Jing Wu, Nan Li, Jia-Jia Du, Jia-Chang Sun, Ting-Ting Chen, Wei Wei, Wu-Yi Sun

**Affiliations:** Institute of Clinical Pharmacology, Anhui Medical University, Key Laboratory of Anti-inflammatory and Immune Medicine, Ministry of Education, Anhui Collaborative Innovation Center of Anti-inflammatory and Immune Medicine, Hefei 230032, China.

**Keywords:** hepatocellular carcinoma, hepatic stellate cells, tumor microenvironment, β2-adrenergic receptor, β-arrestin2

## Abstract

**Background:** β-arrestin2 and β2-adrenergic receptor (β2-AR) have important roles in malignant tumors, the present study aims to investigate the role of activated β2-AR in hepatic stellate cells (HSCs) during hepatocellular carcinoma (HCC) progression and the regulatory effect of β-arrestin2.

**Methods:** Immunofluorescence and Western blot were used to detect the expression of β-arrestin2 and β2-AR in HSCs of liver tissues from human HCC samples and diethylnitrosamine (DEN)-induced HCC model mice. We next used β-arrestin2^-/-^ mice to demonstrate the regulatory role of β-arrestin2 in DEN mice. The subsets of T cells were quantified by flow cytometry. MTT and wound healing assay were applied to detect the proliferation and migration of cells. Co-immunoprecipitation assay was used to detect the link of β-arrestin2 and β2-AR in HSCs. Effect of β-arrestin2 overexpression on β2-AR downstream signaling pathway was verified by Western blot. The secretion of CCL2 was detected by ELISA.

**Results:** The expression of β2-AR was significantly increased, while β-arrestin2 was decreased in HSCs of HCC tissues. And β-arrestin2 deficiency exacerbates DEN-induced HCC accompanied with increased β2-AR expression. The results of flow cytometry showed that the percentage of activated T cells decreased gradually after DEN injection. β-arrestin2 knockout down-regulated the ratio of activated T cells. *In vitro*, selective activation of β2-AR in HSCs promoted the proliferation and migration of HCC cells. β-arrestin2 overexpression enhanced co-immunoprecipitation of β-arrestin2 and β2-AR in activated HSCs, and decreased its downstream Akt phosphorylation. Akt inhibitor decreased secretion of CCL2 in activated HSCs.

**Conclusion:** Our study demonstrated that β2-AR activation in HSCs induces the proliferation and migration of HCC cells may be through Akt signaling, and this effect appears to be regulated by β-arrestin2.

## Introduction

Hepatocellular carcinoma (HCC) is one of the most prevalent malignancies and the fourth leading cause of cancer mortality globally with an estimated 841,080 new cases and 781,631 deaths in 2018 1. There is an essential role of the tumor microenvironment in modulating the growth, metastasis as well as recurrence of HCC. Liver cancer cells are embedded in a complex tumor microenvironment composed of various types of stromal cells 2, such as fibroblasts, inflammatory cells, and endothelial cells. There is a variety of factors are released by activated hepatic stellate cells (HSCs), including extracellular matrix (ECM), chemokines and transforming growth factors, which present within HCC stroma and tend to promote tumor growth and invasiveness^3^,^4^. Additionally, accumulating studies have showed that activated HSCs promote tumorigenicity of HCC 5,6. Therefore, a better understanding of how the properties of tumor cells are influenced by HSCs could be conducive to the development of a novel therapeutic strategy for HCC.

β2-adrenergic receptor (β2-AR) selectively binds to norepinephrine (NE) and epinephrine on the membrane of effector cells and this process is innervated by most sympathetic postganglionic fibers 7. It is reported that in the patients and the rat model with liver cirrhosis, the activation of β2-AR induced by NE is closely related to the occurrence and development of HCC 8-10. Other studies confirmed that positive β2-AR expression is significantly associated with HCC carcinogenic processes 11. Our previous studies have found that NE activates PI3K-Akt signaling and thus induces HSCs activation, as well as ECM accumulation 12. Despite increasing evidence revealing the supportive function of β2-AR in HCC, the underlying role of β2-AR in regulating HSCs properties remains unknown.

β-arrestin2 is a signal regulatory protein, which is involved in the desensitization and internalization of G-protein-coupled receptor (GPCR) and often regarded as a negative regulator of GPCR 13. Additionally, β-arrestin2 plays a key role in cell proliferation, apoptosis, and inflammatory response through mediating signal transduction of tyrosine kinase receptor and mitogen-activated protein kinase. Our previous studies have found that down-regulation of β-arrestin2 promotes tumor invasion and indicates poor prognosis of HCC 14. β-arrestin2 is rapidly recruited to the activated β2-AR at the plasma membrane after agonist stimulation. β2-AR endocytosis and ubiquitination/degradation requires β-arrestin2 as well 15. Nevertheless, whether β-arrestin2 participates in regulating β2-AR signaling and contributes to the progression of HCC requires further investigation.

In the present study, we investigated the potential role of β2-AR in HSCs during HCC progression, and the regulatory effect of β-arrestin2. The results showed that levels of β2-AR increased, while β-arrestin2 expression was down-regulated in HSCs of HCC tissues. Moreover, β-arrestin2 deficiency aggravated diethylnitrosamine (DEN)-induced hepatocarcinogenesis and down-regulated activation of T cell subsets. Expression of β2-AR and level of phospho-Akt (p-Akt)/Akt were increased after β-arrestin2 deficiency, but the level of phospho-ERK (p-ERK)/ ERK had no changes. Furthermore, non-selective β-AR agonist isoprenaline (ISO) and selective β2-AR agonist terbutaline were used to stimulate HSCs* in vitro*. The results demonstrated that activating β2-AR enhances the activation of HSCs, but β1-AR has no obviously effect. Then, we found that the supernatant of HSCs triggered by terbutaline contribute to the proliferation and migration of liver cancer cells. Furthermore, β-arrestin2 and β2-AR were co-expressed, and overexpressed β-arrestin2 down-regulates β2-AR/Akt signaling and secretion of CCL2 in terbutaline stimulated HSCs. These data suggested that β-arrestin2 acts by down-regulating β2-AR/Akt signaling in HSCs to inhibit the proliferation and migration of HCC cells, and therefore has potential as a treatment target in HCC.

## Materials and methods

### Clinical specimens

All specimens were derived from the Department of Pathology, the First Affiliated Hospital of Anhui Medical University (Anhui, China), which includes liver tissue paraffin specimens from patients with HCC and intrahepatic biliary lithiasis (normal control). Those patients were diagnosed, based on clinical symptoms, laboratory, and radiological examinations as well as pathological confirmation. Fresh HCC specimens from patients with HCC and healthy liver tissue from those with hepatolithiasis were formalin-fixed, paraffin-embedded, and cut into 4 μm sections. The present study was approved by the Research Ethics Committee of Anhui Medical University and written informed consents were provided by all patients. The study was performed in accordance with the guidelines established by the Science Council of China.

### Animals

β-arrestin2^-/-^ mice on the C57BL/6J background were purchased from the Jackson Laboratory, their corresponding control wild-type (WT) mice were age- and sex-matched littermates. Each mouse was genotyped at the age of 14 days 16. All mice were maintained under specific-pathogen-free conditions with a temperature of (24±2) °C, a humidity of 50%±10% under a 12 h/12 h dark/light cycle, with free access to water and food. These animal experiments were performed according to protocols approved by the Ethics Review Committee for Animal Experimentation of the Institute of Clinical Pharmacology, Anhui Medical University.

β-arrestin2^-/-^ mice and WT mice were divided into normal and model mice, respectively. HCC model was induced by intraperitoneal (i.p.) injection with one dose of DEN (Sigma-Aldrich, St. Louis, MO) at 20 mg·kg^-1^ body weight, while the normal mice received an equivalent volume of sterilized saline instead 17. After 40 weeks, all mice were performed euthanasia sacrificed after collecting blood. A portion of liver tissues was fixed in 10% formaldehyde solution used for histological analyzes, remaining parts were immediately stored in a refrigerator at -80 °C for subsequent analysis.

### Cell lines culture and treatment

The human HSC line LX-2 and the human liver cancer cell lines HepG2 and SMMC-7721 were purchased from the Institute of Biochemistry and Cell Biology, Chinese Academy of Sciences (Shanghai, China). All cell lines were cultured at 5% CO_2_ with 37 °C in a humidified atmosphere using Dulbecco's modified Eagle's medium (DMEM) (Gibco, USA) containing 10% fetal bovine serum (FBS, Zhejiang Tianhang Biotechnology Co., Ltd, China).

Four groups of LX-2 cells were cultured for future use: LX-2 cells without any treatment; LX-2 cells treated with ISO (Shanghai Harvest pharmaceutical Co., Ltd, China); LX-2 cells treated with β1-AR selective antagonist CGP20712A (Sigma-Aldrich), followed by ISO stimulation; and LX-2 cells treated with β2-AR selective antagonist ICI118,551 (Sigma-Aldrich). In addition, we chose selective β2-AR agonist terbutaline (Sichuan Huayu Pharmaceutical Co., Ltd, China) stimulating LX-2 to further confirm the effect of β2-AR in activated LX-2 cells. To determine which of Akt and ERK signaling has effect on CCL2 secretion, Akt inhibitor LY294002 (MedChemExpress, New Jersey, USA) and ERK inhibitor U0126 (Merck Millipore, USA) were used.

### Liver histopathology

For histological examination, a portion of the left lateral liver tissues was collected from normal mice and HCC model mice, and then fixed in 10% neutral buffered formalin, respectively. After embedded in paraffin, the samples were sectioned at 4 μm thickness. The sections were then routinely stained with hematoxylin for 5 min at 40 °C and eosin solution for 1 min at room temperature. Finally, the microscope was used to evaluate conventional morphological and images were captured at ×200 and ×400 magnification.

### Immunofluorescence double staining

Immunofluorescence double staining was performed to observe the expression of β2-AR and β-arrestin2 in activated HSCs of HCC. The liver tissue and cell slides were incubated with primary antibodies for 12 h in a dark, humid chamber at 4°C. Antibodies against β2-AR (sc-81577), β-arrestin2 (sc-13140) and alpha-smooth muscle actin (α-SMA) (sc-130617) were obtained from Santa Cruz Biotechnology (CA, USA). The next day, slides were incubated with secondary antibodies: goat-anti-rabbit IgG and donkey-anti-mouse IgG (Life Technologies, New York, USA) at room temperature for 1 h in the dark. Following washing 3 times with 1× PBS, DAPI (Zhongshan Goldenbridge Biotechnology Co., Ltd, Beijing, China) was used to stain the nuclei at room temperature for 10 min, and stored at 4 °C. Finally, Leica TCS SP8 confocal microscope (Leica Microsystems, Leica, Germany) was used to observe and capture fluorescent sections.

### Flow cytometry analysis

The splenocytes were transferred to RPMI 1640 medium for cell culture to reach a density of 1×10^7^ cells/mL. To measure the percentage of naive T cell and activated T cell, a total of 200 μL cell suspension of each specimen was incubated at 4 °C in the dark for 30 min after adding CD4-FITC (eBioscience, CA, USA) and CD62-PE (Miltenyi Biotec, NRW, Germany), CD4-FITC and CD69-PE (Miltenyi Biotec) antibodies, respectively. Then the supernatant was removed and cells were resuspended using 1× PBS. To detect the Treg cells proportion, the splenocytes were stained with CD4-FITC and CD25-APC (eBioscience) for 20 min. Then the cells were fixed, permeabilized and stained with labelled Foxp3-PE antibody (eBioscience). To evaluate the percentage of Th17 cells, splenocytes were stimulated with a cell stimulation kit (BD Pharmingen, CA, USA) for 6 h. Cells were stained with CD4-FITC for 20 min at room temperature, then followed by a working fixation solution and permeabilization buffer. Intracellular staining was incubated with IL-17-PE antibody (eBioscience) for 20 min at room temperature. The flow cytometry analysis was performed using a CytoFLEX flow cytometery (Beckman Coulter, Inc., CA, USA).

### Preparation of conditioned medium (CM) of LX2

To detect the effect of LX-2 stimulated with terbutaline on liver cancer cells, the CM of LX-2 cells was prepared. LX-2 cells were seeded in 6-well culture plate at a density of 1×10^6^ cells/mL and incubated at 37 °C in 5% CO_2_. Terbutaline was added at different concentrations (10^-8^-10^-4^ mg·mL^-1^) for 24 h. Following the aforementioned treatments, the medium was replaced with serum-free fresh medium and incubated for 2 days prior to the collection of CM. Cell debris from the supernatants was removed by centrifugation, and the CM was frozen at -80 °C until use.

### Proliferation assay

An MTT assay was performed to investigate the proliferation of LX-2 cells under different condition and liver cancer cells cultured with CM from LX-2 stimulated with terbutaline. All cells were plated in 96-well culture plate in cell incubator overnight. The medium in each well was replaced with serum-free fresh medium and incubated for 12 h. Cells were subsequently with MTT solution (5 mg·mL^-1^, 20 μL/well) at 37 °C for 4 h. Following the treatments, the formazan crystals were dissolved in DMSO (150 μL/well). And then agitating for 10 min on a horizontal oscillator, the plates were analyzed on an Infinite M1000 PRO microplate reader (Tecan Group Ltd., Männedorf, Switzerland). The proliferation of LX-2 and HCC cells were analyzed by calculating the OD value at 490 nm.

### Migration assay

A wound-healing assay was used to examine the migration rate of LX-2 under different condition and HCC cells cultured with CM from LX-2 stimulated with terbutaline. LX-2 and HCC cells were plated in 6-well plates and incubated until 80%-90% confluence was achieved in starvation medium. A “scratch” was created by wounding the cell monolayer with a 200μL-pipette in a straight line. Subsequently, the cells were washed 3 times with 1× PBS and cultured in fresh medium supplemented with 0.5% FBS, followed by incubation with different stimulant from each group. An inverted microscope (IX71; Olympus Corporation, Tokyo, Japan) was used to capture images at 0 and 24 h after incubation. Cell migration was determined by detecting the movement of cells into the scraped area, and quantitative analysis was evaluated using ImageJ software (NIH, Bethesda, MD, USA) with the mean percentage wound closure area relative to the area of the initial wound.

### Overexpressing β-arrestin2 in LX-2 by plasmid transfection

For transient wild-type β-arrestin2 expression, we used a pcDNA3 expression plasmid encoding pEGFP-C2-β-arrestin2, which was kindly provided by Dr. Yang K. Xiang of the University of California, Davis. LX-2 cells were plated in six-well plates and transfected transiently with pcDNA/β-arrestin2 vector using Lipofectamine 3000 (Invitrogen Life Technologies, CA, USA), according to the manufacturer's recommendations. Subsequently, the supernatant in each well was replaced with fresh medium. Cell debris from the supernatant was removed by centrifugation, Western blot was used to confirm β-arrestin2 overexpression successfully.

### Western blot analysis

In general, the proteins of HCC liver tissues and LX-2 cells were extracted using RIPA (Beyotime Biotechnology, Shanghai, China) protein extraction reagent supplemented with phenylmethylsulfonyl fluoride (PMSF, Beyotime Biotechnology) (99:1) following by centrifuged at 12,000×*g* for 10 min at 4 °C, and BCA protein quantitative kit (Thermo Fisher, MA, USA) was used to determine protein concentration. The proteins were separated using 10% SDS-PAGE and then electrotransferred onto PVDF membranes (Millipore, Massachusetts, USA). After transferred, the membranes were blocked with 5% skimmed milk and then incubated at 4 °C overnight with preliminary antibodies. Antibodies against ERK (#9102S), p-ERK (#9101S), Akt (#4691S) and p-Akt (#4058S) were purchased from Cell Signaling Technology (MA, USA). Anti-β-actin (TA-09) was obtained from Zhongshan Goldenbridge Biotechnology Co., Ltd. Subsequently, the proteins were further incubated with horseradish peroxidase (HRP)-conjugated secondary antibodies: goat-anti-rabbit IgG and goat-anti-mouse IgG (Zhongshan Goldenbridge Biotechnology Co., Ltd) for 2 h. The ImageQuant LAS 4000mini imaging system (GE Healthcare Bio-Sciences AB, Uppsala, Sweden) was used to reveal the results. Protein expression levels were defined as grey values, which were determined with ImageJ software version 1.4.2b (NIH, Bethesda, MD, USA). β-actin expression was used as an internal control.

### Enzyme linked immunosorbent assay (ELISA)

To determine the production of CCL2, the supernatants of LX-2 cells were collected and tested in strict accordance with ELISA kit (CUSABIO, Wuhan, China). The standard curve was generated using standard protein provided by the manufacturer.

### Co-immunoprecipitation assay

Cells were collected in RIPA lysis buffer (Beyotime Biotechnology) supplemented with a mammalian protease inhibitor mixture (Biocolors, Shanghai, China). The cell lysate was immunoprecipitated (IP) with anti-β2-AR antibody at 4 °C overnight, subsequently separated by SDS-PAGE and subjected to Western blot analysis with anti-β-arrestin2 antibody. The assay was performed in accordance with standard procedures.

### Statistical analysis

All the results are presented as means ±SD. Pairwise comparisons among groups were conducted by one-way analysis of variance (one-way ANOVA) test. Statistical analysis was performed with the statistical package SPSS 16.0 (SPSS, Inc., Chicago, IL, USA). *P<*0.05 were considered statistically significant.

## Results

### Expression of β2-AR was increased in HSCs of HCC

**Figure 1:**
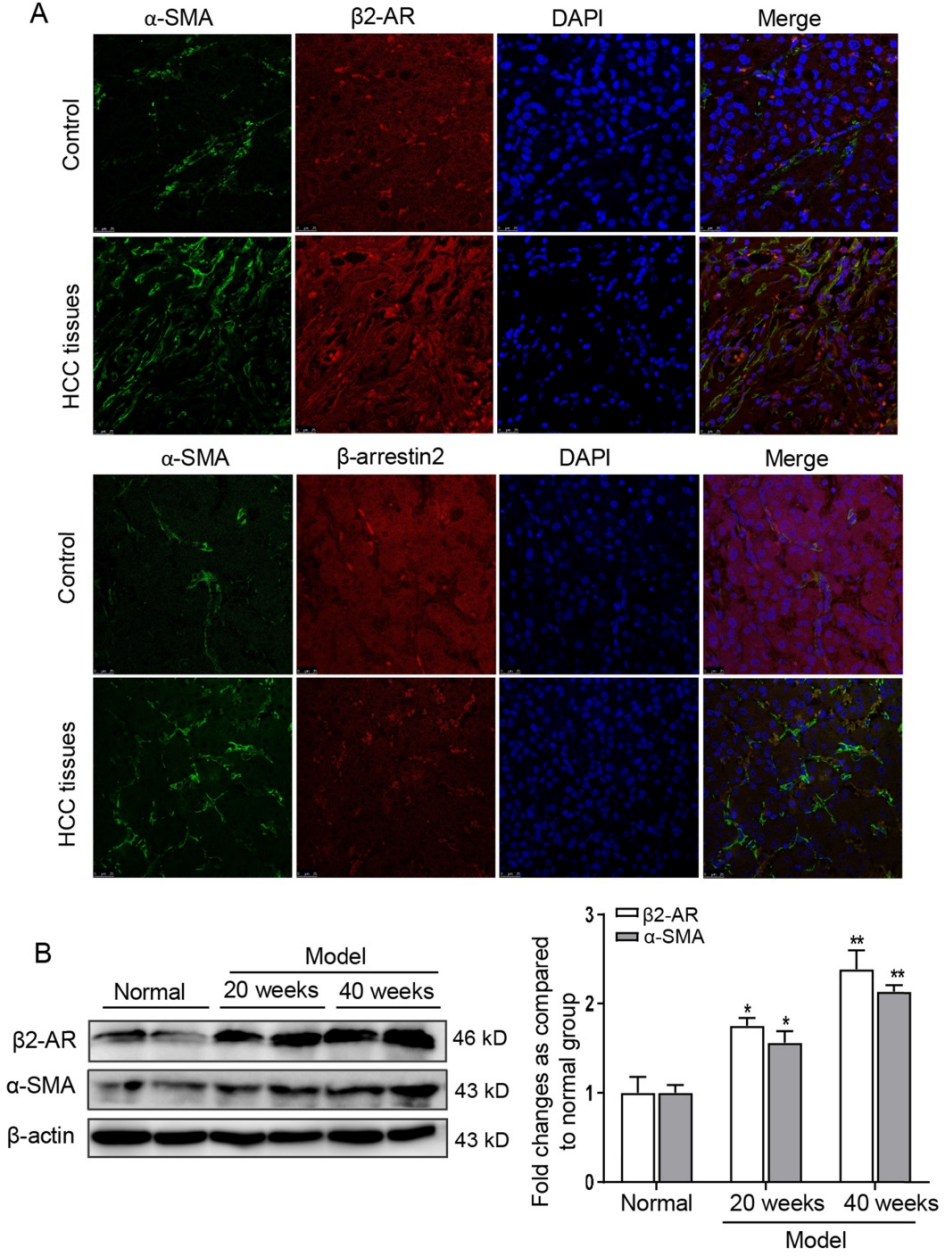
** β2-AR expression is frequently up-regulated in the liver of HCC. (A)** Immunofluorescence analysis of β2-AR and β-arrestin2 expression in activated-HSCs of patients with HCC. The activation of HSCs was represented by α-SMA. Nuclei was stained with DAPI. **(B)** Time course analysis of β2-AR and α-SMA in DEN-induced HCC mice. ^*^P<0.05, ^**^P<0.01 compared with normal mice.

To observe the expression of β2-AR and β-arrestin2 in liver tissues of patients with HCC, β2-AR-positive and β-arrestin2-positive expression appeared as red fluorescent foci, α-SMA (an activated HSCs marker)-positive expression appeared as green fluorescent foci. The fluorescence intensity of α-SMA and β2-AR in liver tissues of HCC patients were stronger than that in hepatolithiasis. However, β-arrestin2 was almost negative staining in HCC patients (Figure 1A). To investigate β2-AR expression and activation of HSCs during HCC progression, we established DEN-induced HCC mice model. At 40 weeks after DEN injection, the protein expression of β2-AR and α-SMA were higher in the liver than 20 weeks post DEN injection (Figure 1B). These data suggested that activated HSCs were up-regulated in HCC patients and DEN-induced liver tumor mice accompanied with increased levels of β2-AR and diminished expression of β-arrestin2.

### β-arrestin2 deficiency aggravated DEN-induced HCC development in mice

**Figure 2:**
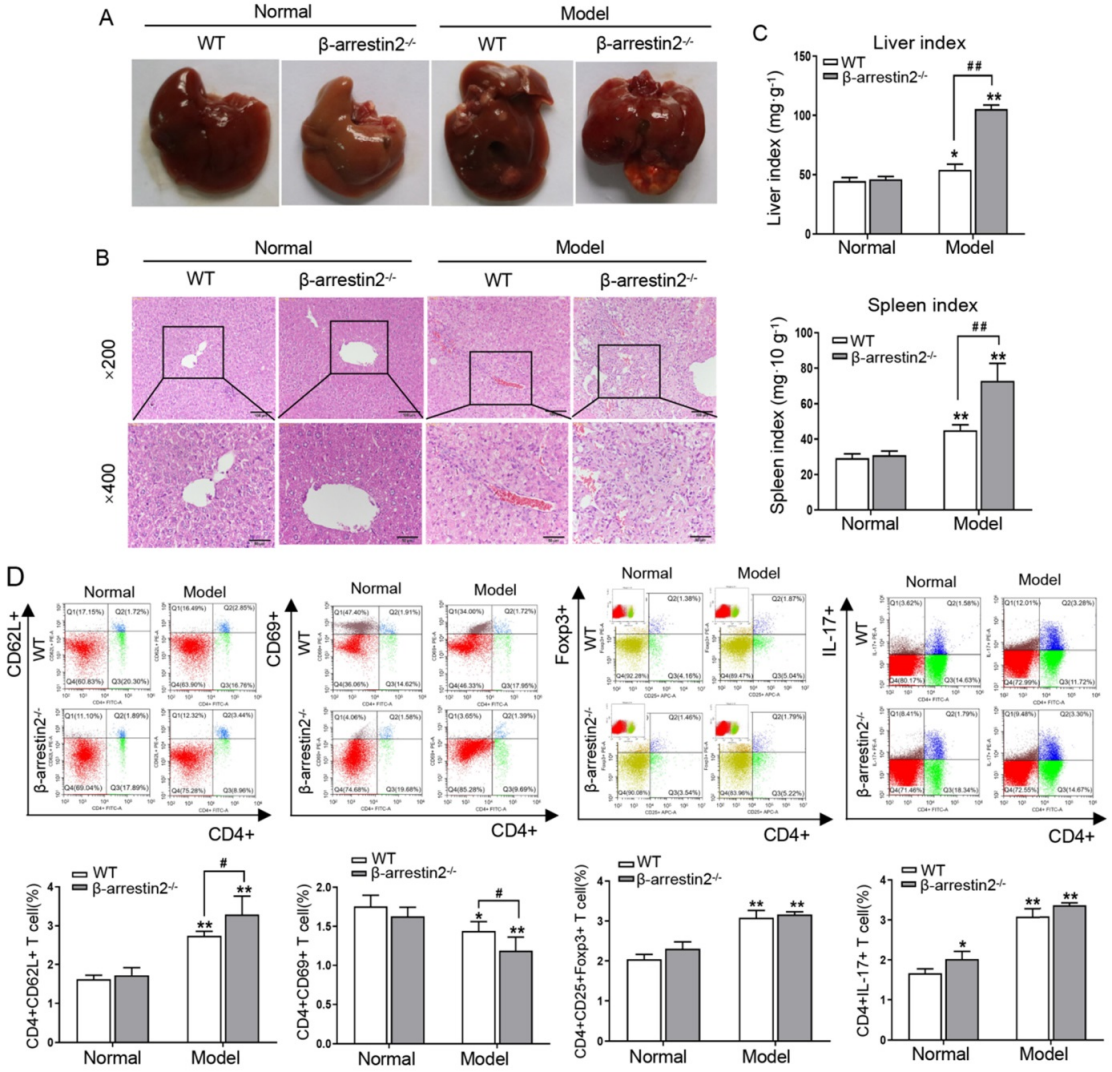
** Loss of β-arrestin2 exacerbates the development of DEN-induced HCC. (A)** β-arrestin2 ablation aggravates the progression of DEN-induced HCC mice. Representative pictures of liver tissue samples from normal mice and β-arrestin2^-/-^ mice. **(B)** Representative photographs of H&E staining from the livers of normal mice and β-arrestin2^-/-^ mice (×200 and ×400 magnification). **(C)** Liver index and spleen index. Liver index (mg·g^-1^) = liver weight (mg)/body weight (g); spleen index (mg·10 g^-1^) = spleen weight (mg)/(body weight (g)×10). **(D)** β-arrestin2 deficiency plays an important role in T cell subsets. Cells were isolated from spleen tissues in each group of mice. The percentage of naive T cells, activated T cells, Treg cells and Th17 cells were determined using flow cytometry and were quantified. Data represent the means ± SD. ^*^P<0.05, ^**^P<0.01 compared with normal mice; ^#^*P*<0.05, ^##^*P*<0.01 compared with WT model mice.

To determine the contribution of β-arrestin2 to HCC, β-arrestin2^-/-^ mice were employed to establish DEN-induced liver tumor model for 40 weeks. As shown in Figure 2A, significant increases in liver size were observed in model mice. Additionally, color changes were noted in DEN-treated livers, as they became more inhomogeneous, spottier and paler than normal control livers. β-arrestin2 deficiency resulted in an increase of prominent macroscopic nodules and larger tumors. The livers of normal mice demonstrated anastomosing plates of hepatocytes radiating from the centrilobular venule towards the periphery of the hepatic lobule. The liver sections exhibited intra-lobular inflammatory cell infiltration, hepatocyte injury and degeneration after DEN injection. β-arrestin2^-/-^ model mice exhibited more severe hepatocyte architecture loss, and extensive vacuolation was noticed in the cytoplasm with masses of acidophilic material (Figure 2B). The liver and spleen indexes from β-arrestin2^-/-^ model mice were significantly increased compared with that from WT model mice (Figure 2C). These data suggested that greater mouse liver tumorigenesis is obviously associated with loss of β-arrestin2 expression.

To determine if loss of β-arrestin2 alters T cell subsets, the subsets of naïve T cells (CD4+CD62L+), activated T cells (CD4+CD69+), Treg cells (CD4+CD25+Foxp3+) and Th17 cells (CD4+IL-17+) were assayed. The proportion of naïve T cells, Treg and Th17 cells enhanced clearly in the spleen of WT model mice. β-arrestin2 deficiency resulted in the increase of naïve T cells and the decrease of activated T cells after DEN injection, but the ratio of Treg and Th17 cells had no significant difference (Figure 2D). These data indicated that β-arrestin2 deficiency may play an important regulatory role in HCC by decreasing the proportion of activated T cells.

### β-arrestin2 knockout enhanced β2-AR expression and downstream signaling in DEN-induced HCC mice

**Figure 3:**
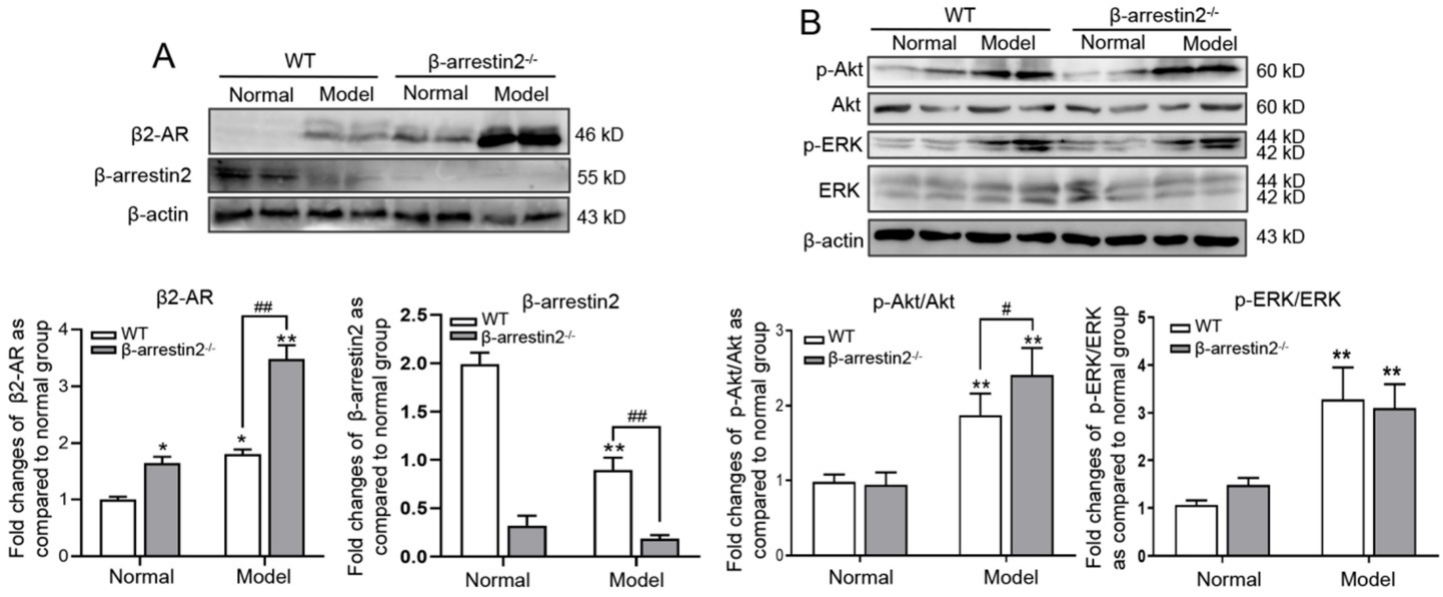
**β-arrestin2 deficiency elevated β2-AR downstream signaling in DEN-induced HCC mice.** Liver homogenate was immunoblotted for β2-AR, ERK and Akt. Representative pictures of the bands from β2-AR **(A)**, the ratio of p-ERK/ERK and p-Akt/Akt **(B)**. These bands intensity was quantified by densitometry and normalized to β-actin. The densitometry values in the histograms were expressed as fold changes relative to normal mice. ^*^P<0.05, ^**^P<0.01 compared with normal mice; ^#^*P*<0.05, ^##^*P*<0.01 compared with WT model mice.

To explore if the aggravation of HCC is due to increase of β2-AR responsiveness, we observed expression of β2-AR and its downstream signal molecules. The results of Western blot showed that expression levels of β2-AR, p-ERK and p-Akt were significantly higher in HCC livers than in normal livers, while β-arrestin2 expression was down-regulated. β-arrestin2^-/-^ mice had a clear increase in the expression of β2-AR and p-Akt but not p-ERK (Figure 3A, B). These results preliminarily indicated that β-arrestin2 deficiency may promote activation of β2-AR/Akt signaling during the development of HCC.

### Selective β2-AR agonist facilitates the activation of HSCs

**Figure 4:**
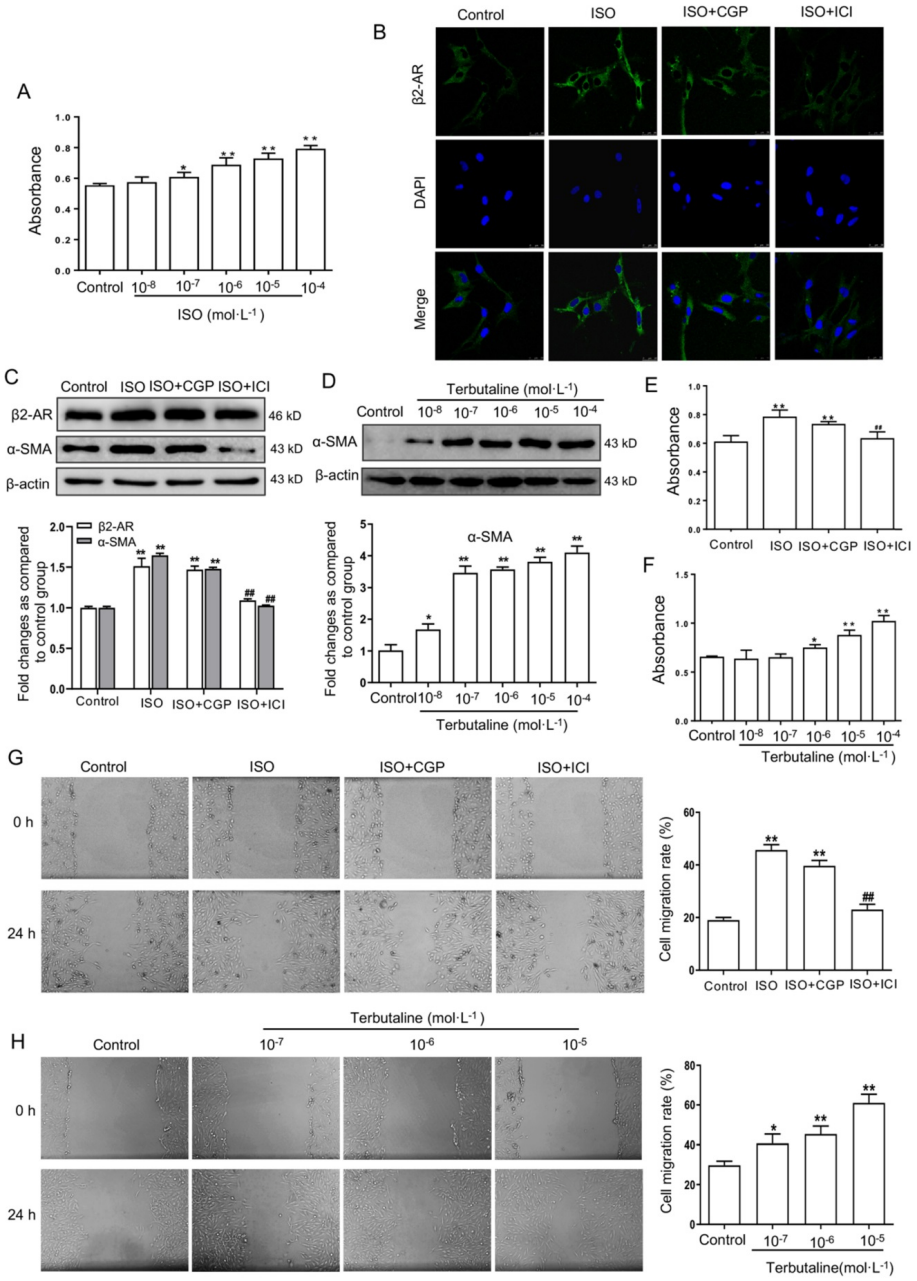
** Activating β2-AR promoted LX-2 cells activation. (A)** The viability of LX-2 cells stimulated by non-selective agonist β-AR ISO (10^-7^-10^-4^ mol·L^-1^) compared with the cells without ISO stimulation by MTT assay. **(B)** Selective β1-AR antagonist CGP20712A and β2-AR antagonist ICI118,551 were used in LX-2 cells stimulated with ISO by immunofluorescence double staining. Antibody against β2-AR (green) were performed. DAPI was used to counterstain the nucleus (blue). **(C)** Western blot assay investigated the role of CGP20712A and ICI118,551 in β2-AR and α-SMA expression in LX-2 cells stimulated with ISO. **(D)** The expression of α-SMA in LX-2 cells stimulated with terbutaline (10^-8^-10^-4^ mol·L^-1^), as presented in the histogram. Data were normalized to the ratio of the control, which was assigned a value of 1 in the graphical presentation. An MTT assay and wound-healing assay investigated the viability and migratory ability of LX-2 cells, respectively. The proliferation **(E)** and migration** (G)** of LX-2 were elevated following activation of β-AR by ISO, and stimulation with ICI118,551 in LX-2 reduced such elevation but CGP20712A had no this effect. Histogram represents the OD (490 nm) of human LX-2 cells. The different concentration of selective β2-AR agonist terbutaline on the proliferation **(F)** and migration **(H)** of LX-2. The histogram represents the fold change in scratch closure of LX-2 cells. ^*^P<0.05, ^**^P<0.01 compared with control cells; ^##^*P*<0.01 compared with ISO stimulated cells.

MTT results showed that the viability of LX-2 cells elevated significantly stimulated by ISO 10^-6^-10^-4^ mol·L^-1^ compared with the cells without ISO stimulation (Figure 4A). Based on the above results, the concentration of 10^-6^ mol·L^-1^ ISO was selected as proper stimulant in the following experiment. The fluorescence intensity of β2-AR in ISO stimulated LX-2 cells were significantly higher than in control cells. β2-AR fluorescence intensity weakened in selective β2-AR antagonist ICI118,551 and ISO stimulated LX-2 cells. However, selective β1-AR antagonist CGP20712A had no significant effect on LX-2 cells stimulated with ISO (Figure 4B).

Given that α-SMA is an activated HSCs marker, it was detected to quantify the activation of HSCs. The results of Western blot (Figure 4C) showed that expression of β2-AR and α-SMA increased significantly in ISO stimulated LX-2 cells. ICI118,551 inhibited the increased expression of β2-AR and α-SMA, but CGP20712A had no this effect. MTT and wound-healing assay showed that ICI118,551 was able to block the increase of cell proliferation and migration by ISO, whereas CGP20712A had no significant effect (Figure 4E, G). To further determine the role of β2-AR in HSCs, LX-2 cells were treated with selective β2-AR agonist terbutaline. Expression of α-SMA was upregulated in LX-2 cells stimulated with terbutaline at the concentrations of 10^-7^-10^-4^ mol·L^-1^ (Figure 4D). The viability and migration of LX-2 cells stimulated with terbutaline (10^-6^-10^-4^ mol·L^-1^) could be significantly elevated compared with the control cells (Figure 4F, H). Altogether, these data suggested that activated β2-AR rather than β1-AR enhances the activation of HSCs.

### β2-AR activation in HSCs significantly enhances the viability and migration of HCC cell lines

**Figure 5:**
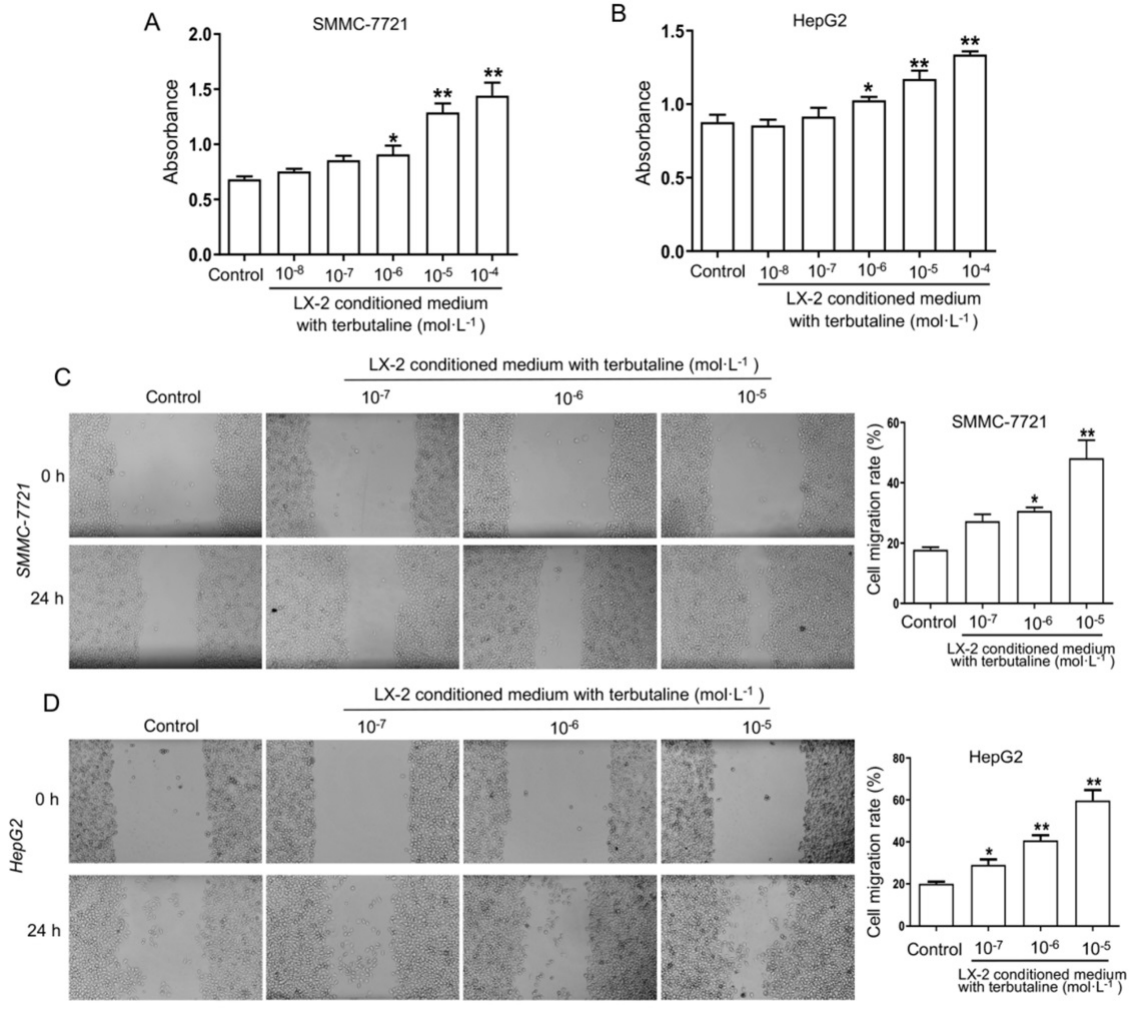
** Terbutaline stimulated LX-2 cells enhanced the viability and migration of HCC cell lines SMMC-7721 and HepG2.** The viability of SMMC-7721 **(A)** and HepG2 **(B)** co-cultured with conditional medium (CM) from LX-2 stimulated by terbutaline respectively. The viability of HCC cells was increased following activation of β2-AR (10^-6^-10^-4^ mol·L^-1^) in LX-2. The migratory ability of SMMC-7721 **(C)** and HepG2 **(D)** cells was investigated by wound-healing assay. Representative photographs are shown. ^*^P<0.05, ^**^P<0.01 compared with control cells.

To investigate the viability and migration of HCC cells is due to β2-AR activation in HSCs, the conditional medium (CM) was obtained from LX-2 stimulated with terbutaline. And then CM of LX-2 was added into human HCC cells SMMC-7721 and HepG2 for co-culture experiment, respectively. The MTT assay results (Figure 5A, B) presented that the viability of SMMC-7721 and HepG2 cells cultured with CM from LX-2 cells activated by terbutaline (10^-6^-10^-4^ mol·L^-1^) were further elevated. Both SMMC-7721 and HepG2 cells cultured with CM of LX-2 cells treated by terbutaline had a higher absorbance at 490 nm. The results of wound-healing assay showed an increasing wound closure and an elevated number of migrated HCC cells co-cultured with CM compared with the control cells at 24 h after wound infliction (Figure 5C, D). These results indicated that the viability and migration attributes of HCC lines co-cultured with CM from terbutaline stimulated HSCs were elevated.

### β-arrestin2 overexpression in activated HSCs impedes β2-AR signaling and secretion of CCL2

**Figure 6:**
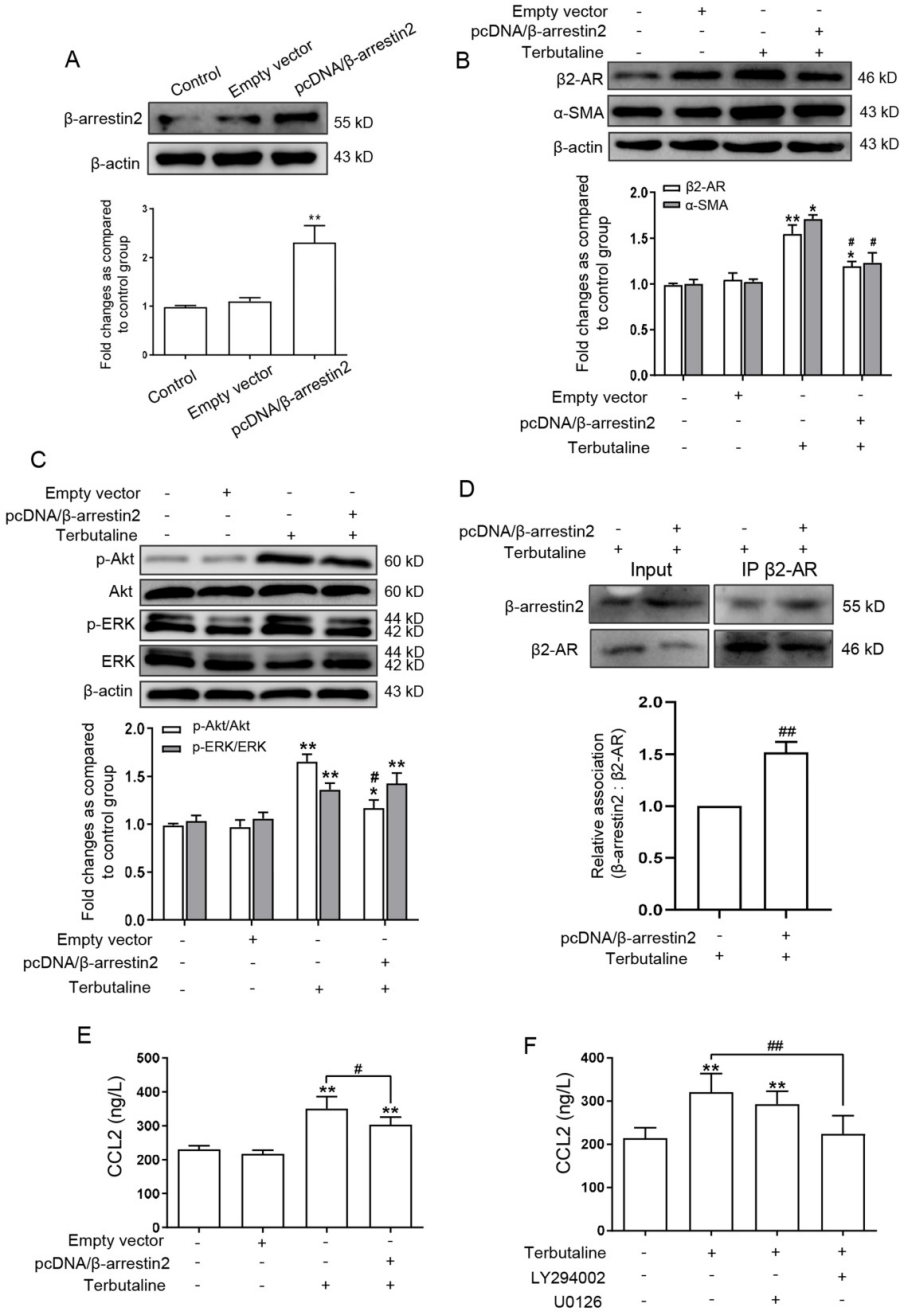
** β-arrestin2 overexpression blocked β2-AR/Akt signaling and reduced CCL2 secretion in LX-2 cells stimulated with terbutaline. (A)** Western blot assay showed that LX-2 cells were transfected by pcDNA/β-arrestin2 successfully. **(B)** Overexpression of β-arrestin2 resulted in the decreased expression of β2-AR and α-SMA in terbutaline stimulated LX-2 cells. The band intensity of β2-AR and α-SMA was quantified by densitometry and normalized to β-actin. Densitometry values in the histograms were expressed as fold change relative to control cells, which was assigned a value of 1. **(C)** β-arrestin2 overexpression inhibited Akt phosphorylation compared with terbutaline stimulated cells but had no effect on ERK. Columns represent the ratios of p-ERK to ERK and p-Akt to Akt. In Western blot assay, gels were run under the same experimental conditions. **(D)** Co-immunoprecipitation experiments of β-arrestin2 and β2-AR in HSCs. ELISA investigated the effect of β-arrestin2 overexpression **(E)**, LY294002 (Akt inhibitor) and U0126 (ERK inhibitor) **(F)** on secretion of CCL2 in terbutaline stimulated LX-2 cells, as presented in the histogram. ^*^P<0.05, ^**^P<0.01 compared with control cells; ^#^*P*<0.05, ^##^*P*<0.01 compared with terbutaline stimulated cells.

It was observed that β-arrestin2 knockout promoted the HCC progression, and activation of β2-AR regulates two major signaling pathways: PI3K/Akt and MEK/ERK1/2, which are involved in the occurrence and infiltration of HCC 18,19. We next postulated that whether β-arrestin2 play a potential regulatory role in HSCs by which β2-AR is regulated. Thus pcDNA/β-arrestin2 plasmid was transfected into LX-2 cells, successful overexpression of β-arrestin2 was confirmed by Western blot assay as shown in Figure 6A. Transfected with pcDNA/β-arrestin2 plasmid significantly inhibited the increase of β2-AR and α-SMA expression stimulated by terbutaline (Figure 6B). To further determine the mechanism by which β-arrestin2 overexpression decreased β2-AR downstream signaling, we measured the expression of p-ERK and p-Akt by Western blot. β-arrestin2 overexpression down-regulated p-Akt in activated LX-2 cells compared with terbutaline stimulated cells. By contrast, p-ERK was detected in all cells overexpressing β-arrestin2 and cells stimulated with terbutaline (Figure 6C). Interaction of β-arrestin2 and β2-AR was observed in HSCs upon terbutaline treatment, as determined by co-immunoprecipitation. While the β-arrestin2/β2-AR interaction was increased after transfected with pcDNA/β-arrestin2 plasmid (Figure 6D). These results suggested that overexpressed β-arrestin2 in HSCs may increase β-arrestin2/β2-AR interaction, thus appearing to inhibit Akt signaling but not ERK.

Previous studies have shown that activated HSCs can promote secretion of CCL2, and the high expression of chemokines CCL2 was detected in HCC patients 20,21. We therefore examined whether CCL2 participates in β-arrestin2 overexpression inhibition of β2-AR signaling. The result of ELISA (Figure 6E) showed that β-arrestin2 overexpression inhibited the high secretion of CCL2 in LX-2 stimulated with terbutaline. To further explore whether CCL2 is associated with β2-AR downstream signaling, we chose LY294002 (Akt inhibitor) and U0126 (ERK inhibitor) to impedes these two pathways, respectively. The results showed that LY294002 negatively regulated secretion of CCL2 in LX-2 stimulated with terbutaline, but U0126 had no this significant effect (Figure 6F). The above data indicated that CCL2 secretion may be regulated by β-arrestin2 overexpression inhibition of Akt phosphorylation in HSCs.

## Discussion

HCC accounts for approximately 90% of primary liver cancers and arises almost exclusively in the setting of chronic inflammation. Induction of liver fibrosis and subsequent cirrhosis often precede hepatocarcinogenesis 22. There are surgical treatment and chemotherapy for HCC patients to relieve pain and improve survival, but the high recurrence rate and prone metastasis that have become a huge obstacle to the eradication of HCC 23,24. Therefore, it is urgent to further explore the characteristics of HCC and to develop the novel therapy.

The tumor microenvironment has been proved to play an important role in modulating the recurrence of cancer 25. HSCs are an important type of cells infiltrated in the tumor microenvironment 26,27. β2-AR belongs to a subtype of ARs, which is permissive for tumor formation and growth. The recent studies have demonstrated that up-regulation of β2-AR was significantly higher in HCC tumor tissues than in their paired nontumorous liver specimens 28. Adrenaline promoted DEN-induced hepatocarcinogenesis, which was reversed by the β2-AR antagonist ICI118,551 29. In our present study, expression of β2-AR abnormally increased in the liver tissues of HCC patients. β2-AR and α-SMA expression were also elevated with increasing severity of model mice. *In vitro*, our results indicated that expression of β2-AR and α-SMA are increased in activated HSCs and CM of terbutaline stimulated HSCs elevate the viability and migration of HCC cells. The data further suggested that β2-AR expression significantly increases activation of HSCs and may promote the development of HCC.

It was showed that in β2-AR-mediated signaling pathway, the well-established classical Gs-adenylate cyclase-cAMP-PKA induces cellular responses 30. In addition, upon activation of β2-AR, PKA mediates β2-AR phosphorylation activating two major signaling pathways: Gβγ/PI3K/Akt and Ras/Raf/MEK/ERK, which are involved in the occurrence and infiltration of HCC 31-33. Meanwhile, the studies suggested that β2-AR mediated cAMP/PKA/ERK1/2 signaling promotes the growth of liver cancer cells 34. In combination with the current study, the results showed that the ratios of p-ERK/ERK and p-Akt/Akt were significantly higher in DEN-induced HCC mice and in terbutaline-stimulated LX-2 cells. These studies indicated that β2-AR may promote the activation of HSCs via ERK and Akt signaling pathway in HCC.

There is mounting evidence suggests that β-arrestin2 not only serves as a regulatory protein for GPCR signaling pathways, but also mediates desensitization and internalization of various receptors 35,36. β-arrestin2 binds to β2-AR that generated a spatial structural obstruction, which blocks GPCR binding to G protein^37^. Our previous study has showed that loss of β-arrestin2 promotes tumor invasion and indicates poor prognosis of HCC 14. The purpose of present study is to deeply investigate how β-arrestin2 regulates β2-AR signaling both *in vivo* and *in vitro*. DEN-induced HCC provides an ideal animal model for HCC carcinogenesis 38. In the results of HE staining, there were more polymorphic cancer cells and inflammatory cells in the liver of β-arrestin2^-/-^ model mice than that of WT model mice. In addition, increased expression of α-SMA and β2-AR in β-arrestin2^-/-^ mice may be due to enhance Akt phosphorylation and thus induce activation of HSCs in HCC. *In vitro*, the results of co-immunoprecipitation demonstrated that β-arrestin2 overexpression may up-regulate its interaction with β2-AR, thus inhibiting the activation of Akt signaling but not ERK. These data potentially suggested that β-arrestin2 deficiency aggravates HCC, and β-arrestin2 may block β2-AR/Akt signaling in HSCs. However, expression of β-arrestin2 was up-regulated in intrahepatic endothelial cells in BDL rats as well as in hepatic arteries from patients with cirrhosis 39. The discrepant results demonstrated that β-arrestin2 may have different functions in different tissues and cell types. Additionally, it was reported that HCC forms a unique immune microenvironment in the process of development 40. The study elucidated the crucial role of immune cell infiltration and immunotherapy, which might contribute to clinical strategies and clinical outcome prediction of HCC 41. It has been reported that the inflammatory cells such as T lymphocytes activate HSCs, which are the major source of myofibroblasts in the liver 42. Intriguingly, β-arrestin2 plays a role in inflammation and the immune response, β-arrestin2 activated CD4+ T lymphocytes in a mouse asthma model 43. β-arrestin2 is a protective player in DSS-induced colitis through modulation of T-cell activation 44. Consequently, we investigate whether β-arrestin2 regulates the proportion of spleen T cell subsets in DEN-induced HCC mice. Our study showed that the proportion of naïve T cells increased in β-arrestin2^-/-^ mice after DEN-injection. Conversely, β-arrestin2 deletion resulted in a decreased percentage of activated T cells. It was suggested that β-arrestin2 deficiency aggravates HCC may be related to inhibit the activated of T cells and thus affecting immune functions of the tumor.

In tumor microenvironment, activated HSCs acquire the phenotype of myofibroblasts and secrete large amounts of ECM and cytokines to promote the development of tumor 45,46. Chemokines are a class of cytokines. When chemokines combined with the corresponding chemokine receptors, they can recruit immune cells into the tumor microenvironment, participate in ECM deposition and play a key role in the proliferation and metastasis of liver cancer cells 47,48. CCL2, monocyte chemotactic protein-1, is an important member of chemokines CC subfamily 20. Previous studies have showed that activated HSCs can secrete CCL2 in the HCC microenvironment 21. Chemokine receptor 2 expression and responsiveness to CCL2-mediated migration were abolished in β2-AR knockout mice, both of which were rescued by β2-AR re-expression 49. Our experiment showed that the CCL2 was significantly increased in the supernatant of LX-2 cells after the stimulation of terbutaline. However, β-arrestin2 overexpression or Akt inhibitor both reduce the elevated secretion of CCL2 secretion in LX-2 stimulated with terbutaline. These results combined with the above data verified that overexpressed β-arrestin2 may down-regulate the activation of β2-AR/Akt signaling and the secretion of CCL2 in HSCs, and thus inhibiting the proliferation and migration of liver cancer cells.

## Conclusion

Taken together, our present investigation provides strong evidence that β-arrestin2 knockout aggravates DEN-induced HCC and regulates β2-AR/Akt signaling. This study also provides insight into the mechanism of β-arrestin2 regulation in HSCs, which contributes to the progression of HCC. In conclusion, β-arrestin2 in activated HSCs plays an important role in HCC progression and metastasis via activation of β2-AR/Akt signaling and secretion of CCL2. Therefore, targeting on β-arrestin2 regulated β2-AR in HSCs to block the proliferation and migration of liver cancer cells may be a potential therapeutic approach for HCC patients.

## Figures and Tables

**Figure 7:**
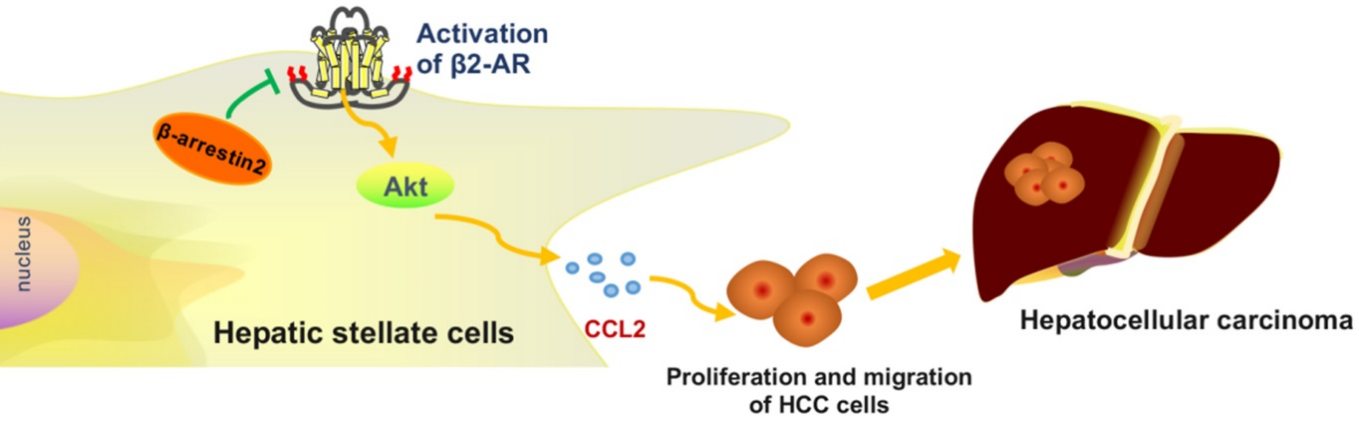
Taken together, the present results combined with our previous data verified that β2-AR/Akt signaling activation in HSCs aggravates the proliferation and migration of HCC cells, β-arrestin2 inhibits β2-AR signaling and thus alleviates the development of HCC.
